# Asymmetry in dentition and shape of pharyngeal arches in the clonal fish *Chrosomus eos-neogaeus*: Phenotypic plasticity and developmental instability

**DOI:** 10.1371/journal.pone.0174235

**Published:** 2017-04-05

**Authors:** Christelle Leung, Kevin Karl Duclos, Thomas Grünbaum, Richard Cloutier, Bernard Angers

**Affiliations:** 1 Department of Biological Sciences, Université de Montréal, Montreal, Quebec, Canada; 2 Département de Biologie, Chimie et Géographie, Université du Québec à Rimouski, Rimouski, Quebec, Canada; Laboratoire de Biologie du Développement de Villefranche-sur-Mer, FRANCE

## Abstract

The effect of the environment may result in different developmental outcomes. Extrinsic signals can modify developmental pathways and result in alternative phenotypes (phenotypic plasticity). The environment can also be interpreted as a stressor and increase developmental instability (developmental noise). Directional and fluctuating asymmetry provide a conceptual background to discriminate between these results. This study aims at assessing whether variation in dentition and shape of pharyngeal arches of the clonal fish *Chrosomus eos-neogaeus* results from developmental instability or environmentally induced changes. A total of 262 specimens of the *Chrosomus eos-neogaeus* complex from 12 natural sites were analysed. X-ray microcomputed tomography (X-ray micro-CT) was used to visualize the pharyngeal arches *in situ* with high resolution. Variation in the number of pharyngeal teeth is high in hybrids in contrast to the relative stability observed in both parental species. The basal dental formula is symmetric while the most frequent alternative dental formula is asymmetric. Within one lineage, large variation in the proportion of individuals bearing basal or alternative dental formulae was observed among sites in the absence of genetic difference. Both dentition and arch shape of this hybrid lineage were explained significantly by environmental differences. Only individuals bearing asymmetric dental formula displayed fluctuating asymmetry as well as directional left-right asymmetry for the arches. The hybrids appeared sensitive to environmental signals and intraspecific variation on pharyngeal teeth was not random but reflects phenotypic plasticity. Altogether, these results support the influence of the environment as a trigger for an alternative developmental pathway resulting in left-right asymmetry in dentition and shape of pharyngeal arches.

## Introduction

The phenotype of an organism is the product of developmental processes and depends on the interactions among genetic, epigenetic and environmental factors. The survival of an individual is intimately dependent on the production of a consistent phenotype according to a specified environmental condition, a capacity defined as developmental stability [1, 2]. Developmental stability is therefore a fundamental characteristic of the development of a given genotype. However, organisms are not impervious to random perturbations and developmental instability refers to the deviation from the expected phenotype within a given environment [3, 4]. Measuring developmental instability represents a proximate way to assess the capacity of organisms to deal with different environments.

While developmental instability can be estimated by measuring the deviation between an observed and expected stable phenotype, this may be a challenging exercise when the optimal or expected phenotype is unknown, which is frequently the case in natural populations [1]. In organisms with bilateral symmetry, the use of bilateral homologous structures across the left-right axis of symmetry offers an accurate way to estimate developmental instability as the development of such structures is expected to be influenced by the same environment and genotype.

However, the influence of the environment during development may result in different outcomes. The environment can be interpreted as a stressor and increase developmental instability (developmental noise [2, 5]), while it may also modify developmental pathways and result in alternative phenotypes (phenotypic plasticity [6]). Left-right asymmetry (L-R asymmetry), the deviation from one side to the other, provides a useful method to discriminate between developmental instability (developmental noise) and environmental influences (phenotypic plasticity) on morphological traits.

Random deviation from L-R symmetry within a given group refers to fluctuating asymmetry (FA) and is often associated to developmental instability [7]. Indeed, FA is seemingly related to accidents during development due to epimutations or abnormalities in the process of ontogenesis [8, 9]. FA is present, at a low degree, in normal development [10–12] but can be increased when an individual is under genetic and/or environmental stresses [13].

On the other hand, directional asymmetry (DA) is a propensity for the trait on one side to develop differently than the other side. Such systematic differences between sides are under precise genetic control and could therefore not be the result of developmental instability. DA results from the particular embryonic development of a given species [14] or a side-specific response to environmental conditions [7]. Consistent phenotypic changes in response to a given environmental condition are referred to as phenotypic plasticity and this process is mediated by epigenetics-environment interactions [15–17].

Fish teeth display a remarkable disparity in size, shape, number, as well as types and structures of attachment to tooth bearing arches or structures [18, 19]. Freshwater cypriniform fishes have lost all dentition on the mandibular arch, while the seventh pharyngeal arch (the fifth branchial arch) has been modified into a tooth bearing pharyngeal jaw apparatus [20]. Dental formulae, referring to the number and arrangement of teeth on the pharyngeal arch, are important taxonomic characters for cyprinid fishes [21]. This character is generally stable within a given species and alternative dental formulae have been traditionally viewed as a consequence of developmental accidents during ontogeny [22, 23].

Fishes typically exhibit bilateral symmetry although internal organs, such as the brain, heart, pancreas and gut, show L-R asymmetry [24]. Several factors are required for proper asymmetric patterning of the vertebrate embryo. In zebrafish, asymmetric development of the brain, heart and gut include monociliated cells within Kupffer’s vesicle, and expression of fibroblast growth factors, retinoic acid, and *wnt11* genes [25, 26]. These factors also appear to be involved in asymmetric craniofacial development and lateralisation of the pharyngeal skeleton in zebrafish [26]. Shape asymmetry of pharyngeal arches in the zebrafish results most frequently in modifications on the R-side [26]. This trend is frequently observed in dental formulae. When asymmetry occur in teeth number, the missing tooth is always on the R-side leading to a higher tooth number on the L-side [27, 28].

This study aims at assessing whether variation of the dentition and the shape of pharyngeal arches result from developmental instability or phenotypic plasticity. We determined the variation in dental formula as well as the shape of pharyngeal arch using X-ray microcomputed tomography (micro-CT scan). A growing body of research has shown the relevance of micro-CT scan and 3D imaging technology to ecological and evolutionary researches [29–31]. This technology has become a cornerstone of the study of tooth morphology and development [32] because it allows a greater precision and incurs less physical loss compared to traditional methods [29]. Pharyngeal arches measure a few millimeters and teeth are very fragile, specimens were thus micro-CT scanned in order to observe tooth positioning and shape variation *in situ*. We estimate FA and DA based on the geometric shape of pharyngeal arches to discriminate whether variation was linked to developmental instability or rather phenotypic plasticity.

This study focuses on the clonal hybrid fish *Chrosomus eos-neogaeus* (Cyprinidae; Teleostei). The absence of genetic variation among individuals makes this asexual fish an ideal system to discriminate the effect of environmentally-induced from those associated to stochastic processes [33]. Clonal fish *Chrosomus eos-neogaeus* resulted from hybridization between northern redbelly dace, *Chrosomus eos*, and finescale dace, *Chrosomus neogaeus*. These all-female hybrids reproduce asexually by gynogenesis; there is neither chromosome segregation nor recombination, leading to genetically identical offspring [33–38]. Therefore, a given genotype can be represented by multiple individuals found in heterogeneous environmental conditions [33, 34]. Moreover, multiple hybridization events led to multiple clonal lineages in distinct sites marked by contrasting environmental conditions [38].

Previous studies reported that combination of different genomes in hybrids may result in higher developmental instability than observed in parental species [39, 40]. One can therefore expect a higher FA in hybrids than in parental species as well as a similar level of variation in dental formulae among sites. On the other hand, if hybrids are more sensitive to a given environmental signal or respond to a lower threshold than parental species, FA is expected to be similar in hybrids and parental species while both shape and dental formula are expected to vary according to site. In addition, DA will reflect phenotypic plasticity when coupled to given phenotype or environmental conditions.

## Material and methods

### Specimen sampling and identification

This research was performed under institutional animal care guidelines (permit #13–084 delivered by the Université de Montréal) and conforms to the mandatory guidelines of the Canadian Council on Animal Care. Sampling permits were provided by the Quebec Ministry of Natural Resources and Wildlife (MRNF).

A total of 262 specimens of the *Chrosomus eos-neogaeus* complex were analysed from 12 natural sites (S1 Table). The different biotypes were identified visually according to external morphological characteristics [41] and confirmed using genetic markers according to Binet and Angers [35]. Specimens included 77 individuals of the paternal species *C*. *eos* from seven geographical localities, 32 individuals of the maternal species *C*. *neogaeus* from a single locality and 153 individuals of the *C*. *eos-neogaeus* hybrids from 12 localities. The assignment of hybrid individuals to a given lineage was achieved using the multilocus genotypes of eight hypervariable microsatellites loci as described in Vergilino *et al*. [38]. A total of eight different hybrid lineages have been analysed (Table 1).

**Table 1:** Characteristics of the individuals analysed in this study.

Site	Latitude	Longitude	*C*. *eos*	*C*. *neogaeus*	Hybrids	Lineages	Total
AS-2	45.96619	-74.02876			9	B-01	9
AS-3	45.91480	-74.02802	30 (28)	32 (14)	19 (10)	B-01	81
AS-8	46.08910	-73.95606			9 (9)	B-01	9
AS-13	46.08710	-73.87888	10 (6)		31 (19)	B-01; B-02	41
NO-1	46.13102	-74.44616			9 (9)	B-01	9
NO-5	45.99389	-74.30582			11 (11)	B-01	11
NO-7	45.94379	-74.19381	4		5 (2)	B-01	9
NO-10	45.91693	-74.07292	10 (9)		11 (6)	B-01	21
RI-2	45.04327	-72.36197	6		9	A-11; B-03	15
SF-4	45.23383	-71.90777	8		6	B-06	14
SF-12	45.13015	-71.67275	9		4	A-18	13
SF-13	45.17686	-71.53598			30	A-06; A-07	30
		Total	77 (43)	32 (14)	153 (66)		262 (123)

Sites, geographic location, biotypes, number of individuals analysed and hybrid lineages detected at those sites.

Site names and codes of lineages referred to Vergilino *et al*. [38].

Number in parentheses refers to the number of individuals analysed for shape.

For hybrids, only individuals of lineage B-01 were analysed for shape.

### Micro-CT scan

Pharyngeal arches were visualised using micro-CT scan. Ethanol preserved fishes were scanned with a SkyScan 1173 micro-CT scan (Brucker-MicroCT, 2011, Belgium). For all individuals, settings were kept constant: source voltage = 58 kV, source current = 71 μA, exposure time = 465 ms, 3 averaging frames over 270° with a rotation step of 0.44°. Scanner flatfield was calibrated prior to each scanning session and flatfield correction was activated. Scans were acquired with no filter, medium resolution parameters (1080 x 1080 pixels) and the zoom was set between 17 and 19.9 μm (a standard zoom of 17 μm was used but had to be adjusted for larger specimens).

Projection images were reconstructed with constant parameters using NRecon (Version 1.6.6.0, SkyScan, Brucker-microCT, Belgium). Reconstruction parameters were kept constant: no smoothing, no beam hardening correction and ring artifact correction was kept at 10.

Reconstructed volumes were analysed with the CTAn package (Version 1.11.4.2, SkyScan, Brucker-micro CT, 2011, Belgium), in order to extract pharyngeal arches as volumes of interest for each specimen. Extraction of volumes was performed manually. Extracted volumes were then stacked with CTVox (Version 2.7.0, SkyScan, Brucker-microCT, 2011, Belgium) in order to visualise pharyngeal arches in 3D.

Extracted volumes were further analysed to produce 3D Polygon File Format mesh files (.ply files). Extracted volumes were converted from bitmap format to dicom format files (.DCM files) using SkyScan Dicom converter (Version 2.1, SkyScan, Brucker-micro CT, 2011, Belgium). Dicom files were subsequently loaded within the open-source software Slicer (Version 4.5 [42]).

3D models were rendered from dicom files using the editor module within Slicer and the thresholding algorithm. Generated models were verified individually and in conjunction with CTVox stacked volumes to ensure quality and fidelity. Model generation incurred artifacts such as holes within the pharyngeal arches and, when possible, thresholding was adjusted to reduce artifacts. Artifacts that did not hinder shape analysis or tooth counts were disregarded.

### Counting pharyngeal teeth

The cyprinid dentition is commonly described with a dental formula [21, 43] referring to the number of teeth in the different rows of the pharyngeal arch. North American cyprinids have one or two rows of teeth on each pharyngeal hemi-arch [23]. As an example, a dental formula 1,5–5,1 indicates the presence of one tooth of the minor row and five teeth on the major row of the L-side–then five teeth on the major row and one tooth of the minor row of the R-side. The most frequent and widespread dental formula within a given biotype was considered the basal pattern for that biotype.

Replacement teeth (*i*.*e*., teeth not attached to the pharyngeal bone) were easily distinguished from functional teeth (*i*.*e*., teeth attached to the pharyngeal bone) by rotating 3D reconstructions. By convention, dental formulae take into account only the functional teeth [23]. Teeth are constantly lost and replaced during the lifetime of a fish. Replacement teeth are synthesised and mineralised before they attach to the underlying bone [19]. The presence of teeth was extrapolated based on the presence of a corresponding cavity associated with the loss of a functional tooth and the presence of a corresponding replacement tooth not yet attached to the bone [23].

### Shape of pharyngeal arches

Geometric morphometric analyses of 3D models were used to describe and measure the shape of the seventh pharyngeal arch (the fifth branchial arch), here after referred to as the pharyngeal arches. A total of 123 individuals were analysed (Table 1), including 43 *C*. *eos*, 14 *C*. *neogaeus* and 66 hybrids of the same lineage (lineage B-01, [34, 38]) from seven geographical localities. The presence of a single lineage among multiple localities represents a unique design to control for genetic differences among individuals and to disentangle the environmental effect on the shape of pharyngeal arches.

**Fig 1:**
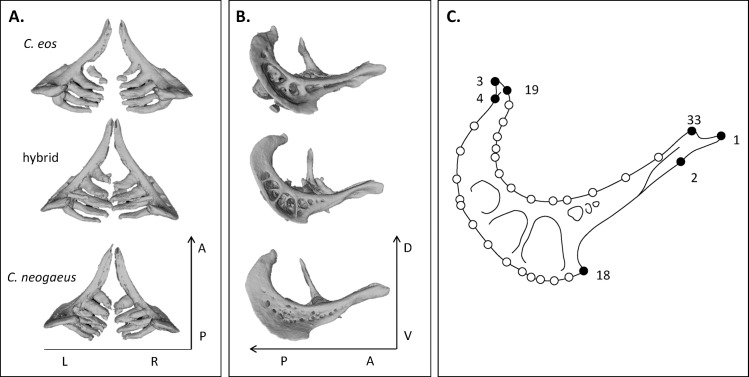
Pharyngeal arches of parental species and hybrids. **A.** Dorsal and **B.** lateral views of the pharyngeal arches. **C.** Landmark scheme for the pharyngeal arches. Filled circles represent landmarks and open circles represent semi-landmarks. Landmarks were positioned on left and right sides of the arch. L: left, R: right, A: anterior, P: posterior, D: dorsal, V: ventral.

Pharyngeal arches (both L and R) of *Chrosomus eos-neogaeus* are rigid crescent-shaped structures displaying an outer curved ridge that is posterior to the arch, and an inner curve dorsal to the arch that delimit the tooth-bearing area. The outer and inner curves represent an important component of the shape of the pharyngeal arch since it covers most of its length. Seven landmarks and 26 sliding semi-landmarks were used to define the shape of both the left and right sides of the pharyngeal arch (Fig 1). Because pharyngeal arches lack bone sutures and foramina, we resorted to Type 2 landmarks which are locally defined points of homology (see [44]). Landmarks were chosen based on their adequacy to describe the overall shape of the arch. Landmarks 1 (anterior-most tip) and 3 (dorsal-most tip) are the endpoints of the arch and serve as anchor points of the structure within the pharyngeal cavity. Sliding semi-landmarks [45] were used to better describe the complex curves of the arch. Thirteen semi-landmarks, delimited by landmarks 4 (change in curvature along the outer ridge) and 18 (anterior limit of the outer ridge), describe the shape of the outer curve of the pharyngeal arch. Thirteen semi-landmarks, delimited by landmarks 19 (change in curvature along the inner curve) and 33 (anterior limit of the inner curve), describe the inner curve of the pharyngeal arch (Fig 1).

### Statistical analyses

The configurations of landmarks were subjected to a Generalised Procrustes Analysis (GPA [46]) to standardize and rotate landmark coordinates. Sliding was performed using the minimum bending energy criterion in order to minimise the deformation between the target shape and the mean shape [47, 48] and to remove the arbitrary variation in the semi-landmark distribution and spacing.

Following landmark superimposition, the R-side was reflected (by multiplying the x coordinates of each landmark and semi-landmark by ‘-1’) to allow landmark correspondence with the L-side. A second superimposition was thereafter performed to align left and right sides and shape variables were extracted from the resulting aligned Procrustes coordinates projected to the shape-tangent space [49, 50].

Landmarks and semi-landmarks were digitised twice for each individual to estimate measurement error due to landmark positioning. The relative amount of shape variation attributable to landmark positioning was assessed using a Procrustes Anova analysis [51] and significance was tested with permutation tests using 999 randomizations.

Phenotypic trajectory analyses [52–54] were used to compare the magnitude and direction of shape differences among *C*. *eos*, *C*. *neogaeus* and *C*. *eos-neogaeus* biotypes for the left and right sides separately.

Individuals were identified according to their biotype (*C*. *eos*, *C*. *neogaeus* or *C*. *eos-neogaeus*), sampled sites and dental formula. Partial redundancy analyses [55] were used to assess the influence of sampled sites and dental formulae on the shape variation of *C*. *eos* and *C*. *eos-neogaeus*. The percentages of the total shape variation that can be attributed to different factors were based on the adjusted R^2^ (R^2^_adj._) [56] and significance of each fraction was tested by permutation tests using 999 randomizations.

Matching symmetry method [57] was used to quantify L-R differences for all biotypes. Procrustes Anova was used to decompose shape variation into variation among individuals, between sides and individual × side interaction [51]. Directional asymmetry was measured using variation between sides and tested using individual × side interaction as an error term. Fluctuating asymmetry was measured using individual × side interaction and tested against measurement error due to landmark positioning.

Statistical analyses were computed with the statistical programming environment R version 3.2.4. We used the *vegan* package (version 2.3–2 [58]) for multivariate analyses and *geomorph* package (version 3.0.2 [59]) for geometric morphometric analyses.

## Results

### Variation in dental formulae

**Fig 2:**
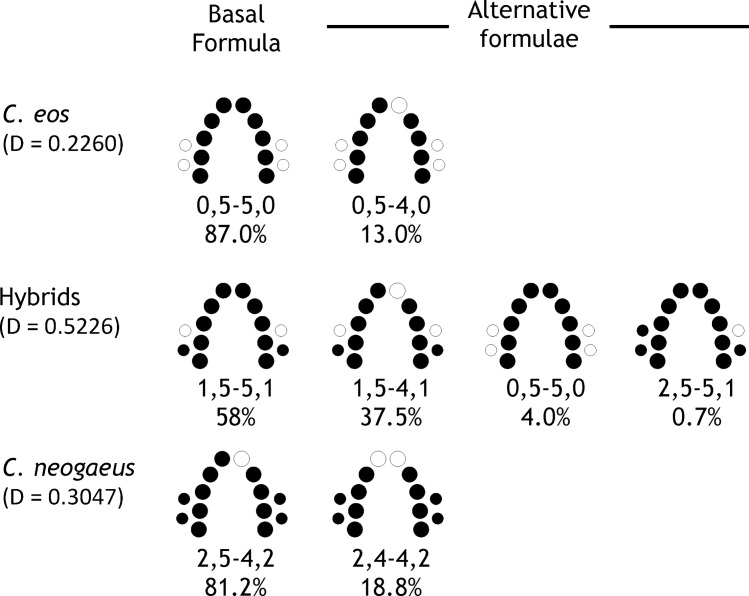
Basal and alternative dental formulae in *Chrosomus eos*, *C*. *neogaeus* and hybrids. Absent teeth in a given formula are indicated by open circle. For each formula, the proportion of individuals from a given biotype is given. The Simpson’s diversity index (D) [63] is provided for each biotype.

A total of seven distinct dental formulae were detected amongst the 262 individuals (Fig 2). Two patterns are present in *C*. *eos* (n = 77). The basal dental formula is symmetric (0,5–5,0) and a single alternative formula (0,5–4,0) was observed in 10 out of 77 individuals. The asymmetric 0,5–4,0 pattern reflects the absence of the first tooth of the major row on the R-side. Two patterns are also observed in *C*. *neogaeus* (n = 32). The basal formula for this biotype is asymmetric (2,5–4,2) and a single alternative formula (2,4–4,2) was observed in 6 out of 32 individuals. The variable position of the alternative formula is the first tooth of the major row on the L-side. Parental species display dental formulae that differ in terms of the number of teeth on the minor arches: zero for *C*. *eos* and two for *C*. *neogaeus*. These results are consistent with previous studies [60–62].

A higher disparity of dental formulae is observed in hybrids than in parental species (Fig 2). The symmetric formula 1,5–5,1 is expected to represent the basal pattern; it is present in all lineages and 58% of the individuals display this formula (Fig 2, Table 2). The 1,5–5,1 formula also requires the fewest changes to adopt alternative configurations, differing from other formulae from only one asymmetric or symmetric change. When compared to parental species, the hybrid basal dental formula displays an intermediate number of teeth on the minor arch (1,5–5,1) as reported by Goddard *et al*. [60] and Binet and Angers [35].

**Table 2:** Dental formulae of the different hybrid lineages. For each lineage, the number of individuals per dental formula is given. Diversity refers to the Simpson’s diversity index.

Site	Lineage	1,5–5,1	1,5–4,1	0,5–5,0	2,5–5,1	Total	Diversity
AS; NO^*^	B-01	45	48			93	0.4995
AS-13	B-02	6	4			10	0.4800
RI-2	A-11	2	4			6	0.4444
RI-2	B-03	3				3	0
SF-4	B-06	6				6	0
SF-12	A-18	3	1			4	0.3750
SF-13	A-06	18			1	19	0.0997
SF-13	A-07	5		6		11	0.4959

^*^ AS-2, 3, 8, and 13; NO-1, 5, 7, and 10

The number of dental formulae, as well as the relative abundance of each formula are higher in hybrids than in parental species; this is reflected in the higher Simpson’s diversity index in hybrids (D = 0.5226) than in *C*. *eos* (D = 0.2260) and *C*. *neogaeus* (D = 0.3047). Two lineages, B-03 and B-06, displayed only the basal formula but we cannot rule out the possibility of alternative formulae for these lineages due to the low sample size (3 and 6 individuals, respectively; Table 1). Each of the other lineages presents two distinct dental patterns and display diversity ranging between 0.375 and 0.4995 (except A-06 D = 0.0997). Two alternative formulae involving variation of the minor row were found: six out of 11 individuals of lineage A-07 display the 0,5–5,0 formula observed in paternal species *C*. *eos* and a rarer formula, 2,5–5,1, found in a single individual out of 19 in lineage A-06.

The most frequent alternative dental formula is asymmetric (1,5–4,1) lacking one tooth on the R-side of the arch as observed on the major row of *C*. *eos*. This pattern is present in four distinct lineages (B-01, B-02, A-11 and A-18) and is represented by 37.5% of the individuals. However, when considering exclusively lineages where this pattern is present, 50.44% of the individuals shared this phenotype indicating the high prevalence of this alternative dental formula.

**Fig 3:**
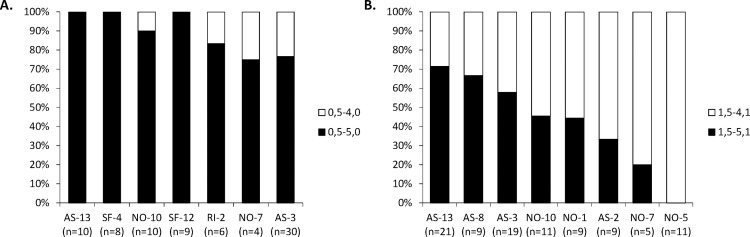
Variation in dental formulae in *C*. *eos* and hybrid lineage B-01 among sites. The relative abundance of basal and alternative dental formulae detected in **A.** *C*. *eos* and **B.** hybrids from each site. Numbers in parentheses indicate sample sizes.

Between 75 to 100% of the *C*. *eos* individuals share the 0,5–5,0 dental formula (Fig 3); it is not correlated to site (R^2^_adj_ = 0.020507; *P* = 0.276). The number of *C*. *eos* individuals with an alternative formula is not significantly different from the one reported by Eastman and Underhill (1973) (11 out of 137 individuals; χ^2^ = 1.3691; 1 df; *P* = 0.242). These results indicate a similar proportion of alternative pattern in *C*. *eos* from different locations.

On the other hand, the abundance of individuals harboring the 1,5–5,1 basal dental formula among clones of the lineage B-01 ranges from zero (N-05) to 70% (AS-13) (Fig 3). Contrasting with the parental species *C*. *eos*, the abundance of the different dental formulae is correlated to sampled sites in B-01 hybrids (R^2^_adj_ = 0.1395; *P* = 0.004). This result indicates a non-random variation in abundance of individuals harboring the 1,5–5,1 formula among lakes.

### Variation in shape of the pharyngeal arches

Procrustes ANOVA analyses confirmed that inter-individual variation was higher than within-individual variation associated to landmark positioning (L-side: R^2^ = 0.8697, *P* = 0.001; R-side: R^2^ = 0.8560, *P* = 0.001). Shape variation is not significantly different between landmark digitising replicates (L-side: *P* = 0.600; R-side: *P* = 0.633). Differences among biotypes and individuals as well as between L-R sides are therefore expected to be higher than the proportion of variation due to error in landmark positioning.

**Fig 4:**
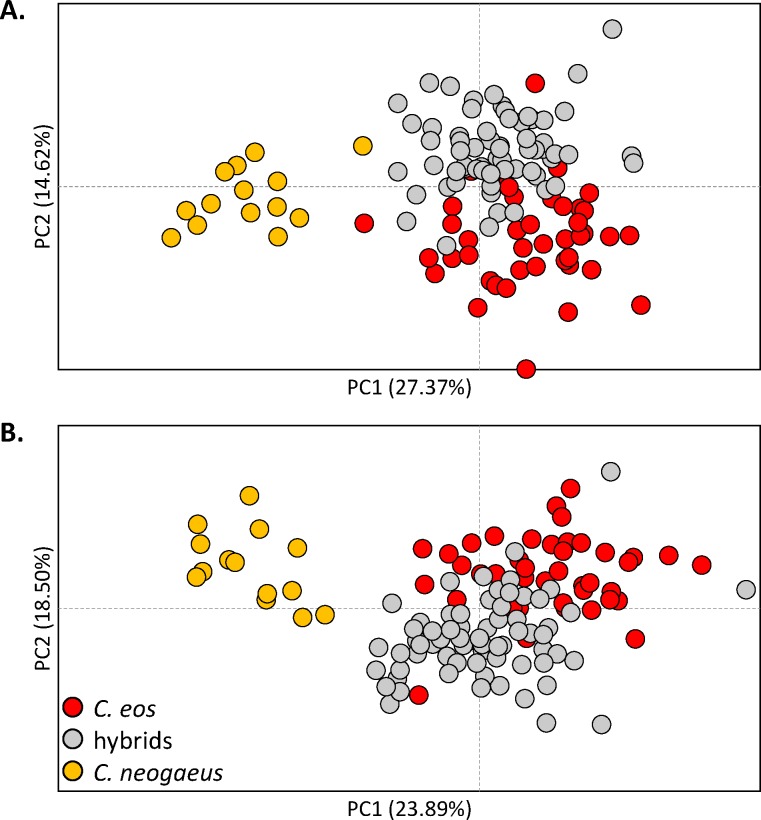
Disparity on shape of pharyngeal arches among the parental species and hybrids. Principal component analysis scatter plot for **A.** the left side and **B.** the right side for *C*. *eos* (red), hybrids (grey) and *C*. *neogaeus* (orange).

Redundancy analysis revealed that the shape of pharyngeal arch is significantly different among the two parental species and hybrids for the left and right sides (Fig 4). However, hybrids are more similar to *C*. *eos* than to *C*. *neogaeus* (Table 3). Trajectory analyses confirmed that the magnitude of phenotypic differences between hybrids and *C*. *eos* is significantly lower than those between hybrids and *C*. *neogaeus* (*P* < 0.001 for both sides).

**Table 3:** Statistical assessment of shape differences among biotypes for the left and right sides of pharyngeal arch.

	Pairwise comparison	R^2^_adj_	*P*_Shape_	*P*_Size_	*P*_θ_
Left	*C*. *eos vs* hybrids	0.1291	< 0.001	a	a
	*C*. *eos vs C*. *neogaeus*	0.3095	< 0.001	b	b
	*C*. *neogaeus vs* hybrids	0.2631	< 0.001	b	b
Right	*C*. *eos vs* hybrids	0.1062	< 0.001	a	a
	*C*. *eos vs C*. *neogaeus*	0.3453	< 0.001	b	b
	*C*. *neogaeus vs* hybrids	0.2559	< 0.001	b	b

Observed significance levels for shape differences (*P*_shape_) and pairwise comparison of trajectory size (*P*_Size_) and angles (*P*_θ_) were generated from 999 random permutations. Pairwise comparisons sharing the same letter are not significantly different (*P* > 0.151).

In contrast to the relative abundance of *C*. *eos* dental formulae that were randomly distributed among sites, the shape of *C*. *eos* pharyngeal arch was significantly different among sites for the L-side (R^2^_adj_ = 0.1274; *P* = 0.001) and R-side (R^2^_adj_ = 0.1398; *P* = 0.001). However, shape variation is not correlated to dental formulae for both L-side (R^2^_adj_ = -0.0031; *P* = 0.591) and R-side (R^2^_adj_ = 0.0053; *P* = 0.261).

For the pharyngeal arches of hybrids, partition of shape variation revealed significant effect of site for both the L-side (R^2^_adj_ = 0.0951; *P* = 0.001) and R-side (R^2^_adj_ = 0.1132; *P* = 0.001). Because relative abundance of dental formulae was detected to be not randomly distributed among sites, a confounding effect may exist between both factors. Partition of shape variation was therefore performed by controlling one or the other factor to take into account confounding effects. Pure site effect remained significant (L-side: R^2^_adj_ = 0.0993; *P* = 0.001; R-side: R^2^_adj_ = 0.1039; *P* = 0.001) indicating that shape of pharyngeal arches was still significantly different among sites, despite shape differences induced by dental formulae. Shape variation was correlated to individual dental formulae for the R-side (R^2^_adj_ = 0.0142; *P* = 0.029) but not for the L-side (R^2^_adj_ = 0.0086; *P* = 0.092). When controlling for the sites, no pure dental formulae effect was detected for the shape of the R-side (R^2^_adj_ = 0.0049; *P* = 0.191). The absence of pure dental formulae effect confirms the confounding effect with site and suggests therefore a co-variation of shape and dental formulae according to environmental conditions.

The difference in dental formulae effect upon shape variation of each side of the arch suggests a possible L-R asymmetry in hybrids. Because hybrids displayed distinct dental formulae among sites, DA and FA were assessed for hybrids by sorting individuals according to their dental formulae (1,5–5,1 *vs* 1,5–4,1) in order to test whether dental formulae may result from an environmental signal that is not site-specific. Because both parental species presented more stable dental formulae compared to hybrids (Fig 2), matching symmetry analyses were also performed on parental species as a control to assess whether asymmetry in shape of pharyngeal arches is caused by the absence of a tooth in the major row. DA and FA analyses were thus performed for parental individuals displaying symmetric (35 *C*. *eos* with 0,5–5,0 formula) and asymmetric (14 *C*. *neogaeus* with 2,5–4,2 formula) dental formulae (Table 4).

**Table 4:** Analysis of shape asymmetry. Results of Procrustes Anova for parental species and hybrids displaying symmetric or asymmetric dental formula.

	Source	df	SS	MS	F	Pr (>F)
*C*. *eos*	Individual	34	0.4094	0.0120	1.3974	0.001
0,5–5,0	Side	1	0.0082	0.0082	2.5882	**0.009**
	Individual × Side	34	0.0922	0.0027	0.9621	0.604
	Measurement error	70	0.1029	0.0014		
*C*. *neogaeus*	Individual	13	0.1024	0.0079	3.3928	0.184
2,5–4,2	Side	1	0.0019	0.0019	0.8012	0.654
	Individual × Side	13	0.0302	0.0023	2.8006	0.099
	Measurement error	28	0.0232	0.0008		
Hybrids	Individual	30	0.2102	0.0070	1.0242	0.355
1,5–5,1	Side	1	0.0040	0.0040	1.1195	0.241
	Individual × Side	30	0.0962	0.0032	1.0221	0.368
	Measurement error	62	0.0972	0.0016		
Hybrids	Individual	34	0.2789	0.0082	0.9046	0.842
1,5–4,1	Side	1	0.0144	0.0144	3.0820	**0.001**
	Individual × Side	34	0.1337	0.0039	1.1968	**0.010**
	Measurement error	70	0.0946	0.0014		

df, degrees of freedom; SS, sums-of-squares; MS, mean square; F, F-statistic

No FA was detected for *C*. *eos* (*P* = 0.604) and *C*. *neogaeus* (*P* = 0.099). No effect of side was detected in *C*. *neogaeus* (*P* = 0.654) in spite of asymmetric dental formulae. An effect of the side (L or R) was detected in *C*. *eos* (*P* = 0.009) but this effect did not remain significant when controlling for sites (*P* = 0.136). In hybrids, FA (*P* = 0.368) and DA (*P* = 0.241) are not significant for individuals with the symmetric 1,5–5,1 dental formula. Lack of L-R asymmetry for the shape of individuals harboring the basal formula suggests a high developmental stability in these hybrids.

On the other hand, FA (*P* = 0.010) is significant for individuals with the asymmetric 1,5–4,1 dental formula, indicating they exhibit a higher developmental instability than hybrids with the symmetric dental formula. Interestingly, in contrast to the shape differences observed among sites, individual side deviation is not significantly different among localities (R^2^_adj_ = 0.0036; *P* = 0.441) and confirms that FA is not associated with environmental conditions of sites. In addition, this lack of correlation indicates that site-specific shape differences observed in pharyngeal arches is not involved in L-R asymmetry. In addition to FA, DA is also highly significant (*P* = 0.001) and remains significant when controlling for sites (R^2^_adj_ = 0.0332, *P* = 0.003). This indicates that hybrids with asymmetric dental formulae also display a marked L-R difference in pharyngeal arch shape regardless of their site of origin.

**Fig 5:**
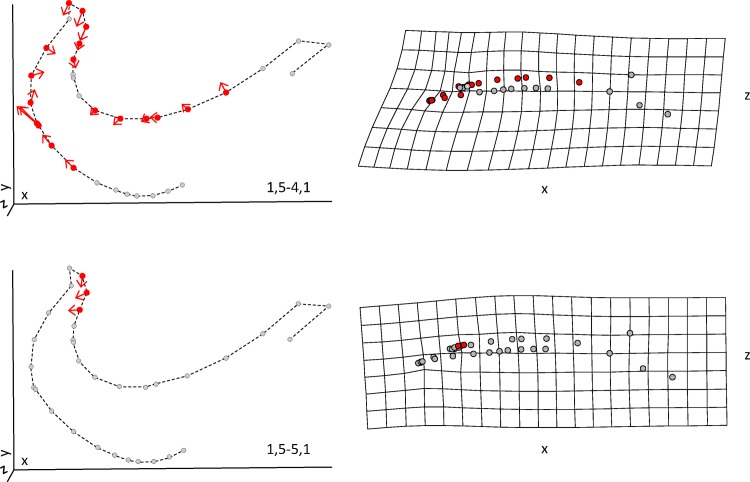
Left-right asymmetry in shape for hybrids. Left panel: landmarks with significant deviation are represented by red circles and a vector (magnified 5×). Right panel: deformation grid of the X and Z axes.

L-R difference was assessed for each landmark (including both landmarks and semi-landmarks) individually by controlling for putative site effect. Only three landmarks (positioned on the anterior margin of the arch) displayed significant asymmetry (*P* < 0.043) with R^2^_adj_ that varied between 0.0312 and 0.0638 for the individuals bearing the 1,5–5,1 formula. On the other hand, analyses performed on individuals with the 1,5–4,1 formula revealed 18 landmarks with a significant L-R asymmetry (*P* < 0.043) with R^2^_adj_ that varied between 0.0228 and 0.0856 (Fig 5). Eleven of these landmarks coincide with the inner margin of the arch where the teeth are attached and where the tooth is lacking on the R-side. The seven remaining landmarks are located on the posterior margin of the arches and indicate a bias toward a thinner structure on the R-side.

## Discussion

Analysis of eight clonal lineages of the hybrid fish *Chrosomus eos-neogaeus* revealed a higher number of dental formulae as well as a higher abundance of each formula in hybrids than in their sexual parental species, *C*. *eos* and *C*. *neogaeus*. The same basal symmetric dental formula (1,5–5,1) is detected regardless of the hybrid lineage. The most frequent alternative dental formulae is asymmetric (1,5–4,1) being present in several distinct lineages (B-01, B-02, A-11 and A-18). These dental formulae have been previously reported by Goddard *et al*. [60] and Binet and Angers [35]. While few studies have been undertaken to examine the intraspecific variation that may occur in dental formula (*e*.*g*. [23]), such variation appears unusual, especially in the absence of genetic difference among individuals.

### Plasticity of the pharyngeal jaw apparatus

The correlation between the different dental formulae of hybrids from the lineage B-01 and sampled sites indicates a non-random variation in the abundance of individuals displaying the symmetric and asymmetric dental formula among lakes. This contrasts with the similar proportion of alternative patterns in the parental species *C*. *eos* among sites indicative of random changes. This non-random variation ruled out that the asymmetric dental formula of clonal hybrids is only the result of ontogenetic accidents as proposed in other species (*e*.*g*. [23]).

A given clonal lineage is not devoid of genetic variation as mutations occur constantly and populations from different sites are often fixed for different alleles [33–35, 38]. Fixation of new alleles leads to the loss of genetic polymorphism because all nucleotides are linked one to each other in absence of recombination (complete lineage sorting). It is thus extremely unlikely that genetic polymorphism be responsible for the morphological variation observed among populations. In addition, the same phenotypic variants have been observed in lineages resulting of distinct hybridization events, with distinct genetic background. Therefore, this relationship between the relative abundance of each dental formulae and the environment is indicative of a reaction norm triggered by environmental influence (*e*.*g*., site dependence).

The shape analysis of the arches also revealed that environmental conditions of each lake influence the shape of both hybrids with symmetric or asymmetric dental formula as well as *C*. *eos*. This suggests that parental species and hybrids display similar level of developmental stability (canalisation) during their respective development.

### Asymmetry in the shape of pharyngeal arches

Dental formulae and arch shape are strongly correlated since DA was restricted to hybrids with asymmetric dental formula. Both asymmetry in shape and dental formulae occurred on the R-side. These results are consistent with previous study on zebrafish [26] for which a R-sided bias of asymmetric and aberrant pharyngeal arch development is observed in *ace/fgf8* mutants. We can however exclude mechanical deformations of the R-side as a consequence of the absence of one tooth because no such DA was detected in *C*. *neogaeus* for which the basal formula is asymmetric (2,5–4,2). Systematic differences between L-R sides do not appear to be the result of developmental instability but a side-specific response to environmental conditions [7]. The L-R asymmetry detected on all individuals with the asymmetric formula 1,5–4,1 suggests an altered developmental pathway affecting both shape and dental formulae in these hybrids, regardless of the site of origin. Consistent discrete phenotypic changes in response to a given environmental signal are referred as phenotypic plasticity, more specifically polyphenism [64]. Such process is triggered by environmental signal and incorporated in the developmental pathway *via* epigenetic processes [15–17, 65].

A portion of L-R asymmetry results from stochastic errors (FA) during development indicating a higher developmental instability in hybrids with an asymmetric dental formula. FA is a good indicator of developmental instability [7, 13]. However, it cannot be associated to a genomic stress alone as all hybrids analysed for shape belong to the same lineage. In addition, FA is not significant in both parental species and hybrids with symmetric dental formula, indicating that the hybrid genome itself did not result in a higher instability during the development of pharyngeal arches. The hypothesis of genomic incompatibilities could then be ruled out at least for the dental formula and pharyngeal arch shape in clonal *C*. *eos-neogaeus* hybrids. FA is not different among sites. This indicated that developmental instability due to site-specific environmental conditions does not explain the variation observed in the different relative proportions of dental formulae among lakes. Therefore, FA detected in hybrids with an asymmetric dental formula likely results from a stressful development associated with the alternative pathway. This system provides an empirical support demonstrating the correlation between phenotypic plasticity and developmental instability. Developmental instability is often recognised as a consequence or more specifically a cost of phenotypic plasticity [66, 67]. However, the lack of studies on the functional performance of asymmetric shape and dental formulae do not allow assessment of fitness variation, if any.

### A threshold character

The results of this study suggest a strong influence of the *C*. *eos* genome on the development of the whole pharyngeal arch in hybrids. With respect to the major tooth row, hybrids and *C*. *eos* display similar basal formula (n,5–5,n) as well as alternative formula (n,5–4,n). In addition, one lineage displays an alternative dental formula identical to that of *C*. *eos* (0,5–5,0). Tooth shape is more similar between *C*. *eos* and hybrids. Teeth are elongated and digitiform in both *C*. *eos* and hybrids, whereas they are more conical with much larger bases and a shorter length relative to the arch in *C*. *neogaeus* (Fig 1A).

The overall shape of pharyngeal arches also supports this trend. For instance, the lateral surface of the pharyngeal bones of *C*. *neogaeus* displayed a punctured texture characterised by small lacunae distributed irregularly, whereas *C*. *eos* and hybrids display large reticulated cavities (Fig 1B). However, reticulations are thinner and more abundant in hybrids than in *C*. *eos*. Finally, the analyses on arch shape revealed that hybrids are more similar to *C*. *eos* than to *C*. *neogaeus*. Trajectory analyses confirm that the magnitude of phenotypic differences between hybrids and *C*. *eos* is significantly lower than those between hybrids and *C*. *neogaeus*.

The lack of environmental influence on dental formulae variation in *C*. *eos* suggests a random process. In addition, the proportion of *C*. *eos* individuals with an alternative formula did not differ from the one reported by Eastman and Underhill [23] from a distinct geographical region. However, the same alternative dental formulae found on the major row in *C*. *eos* and hybrids suggests a shared alternative developmental pathway. Interestingly, *C*. *eos* individuals with an asymmetric dental formulae displayed a significant FA (*P* = 0.032) in spite of a low sample size (n = 7) indicating a higher instability during the development of the arch for the individuals displaying an asymmetric dental formulae. However, we do not detect DA (*P* = 0.209) in these individuals.

**Fig 6:**
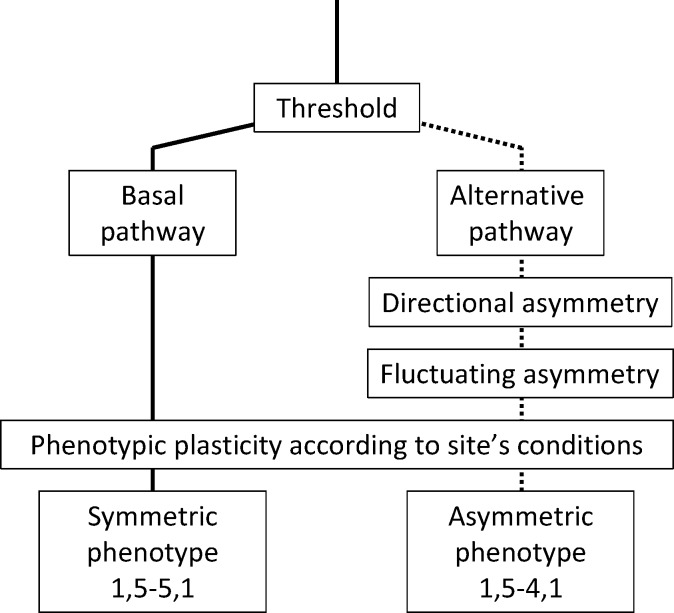
Schematic representation of the hypothetical processes occurring during the development of the pharyngeal arches in clonal hybrids. The alternative pathway is induced when individuals reach a given threshold, resulting in consistent L-R asymmetry in shape and dental formula as well as developmental instability.

Altogether, these results suggest that discrete characters observed in pharyngeal arches could be a threshold character (Fig 6) for which genetic and environmental conditions determine a threshold value above which an alternative developmental pathway will be initiated [68]. Genetic changes in thresholds are known to influence reaction norms [69]. The high variation in dental formulae observed in hybrids and contrasting with the relative stability across species suggests this threshold value could have been influenced by hybridization. Hybrids would be therefore more sensitive to environmental signals than parental species. This alternative developmental pathway results in consistent asymmetric dental formula and arch shape as well as a higher developmental instability.

In conclusion, the results of the present study suggest that variation in dental formulae of the clonal fish *Chrosomus eos-neogaeus* is a consequence of an alternative pathway induced by environmental signal(s) during the development of pharyngeal arches. Variation in dental formulae as well as pharyngeal arch shape of these hybrids would therefore represent a polyphenism, a particular case of phenotypic plasticity, rather than the result of developmental instability. The relative stability of dental formulae reported in multiple species including *C*. *eos* [23] suggests a higher sensitivity for the environmental signal triggering this alternative development pathway in the hybrids. The FA associated to this pathway highlights the correlation between phenotypic plasticity and development instability.

## Supporting information

S1 Table Landmarks and semi-landmarks coordinates raw data. Biotypes, sampling site, dental formula and X, Y and Z coordinates for each landmark and semi-landmark positioning session and side are provide for each individual.
(XLSX)

## References

[1] Møller AP, Swaddle JP. Asymmetry, developmental stability and evolution: Oxford University Press, UK; 1997.

[2] Debat V, David P. Mapping phenotypes: canalization, plasticity and developmental stability. Trends Ecol Evol. 2001;16(10):555–61.

[3] Palmer AR. Fluctuating asymmetry analyses: a primer In: Markow TA, editor. Developmental instability: its origins and evolutionary implications: Springer Netherlands; 1994 p. 335–64.

[4] Palmer AR, Strobeck C. Fluctuating asymmetry: measurement, analysis, patterns. Annu Rev Ecol Syst. 1986;17:391–421.

[5] Waddington CH. Canalization of development and the inheritance of acquired characters. Nature. 1942;150(3811):563–5.

[6] West-Eberhard MJ. Phenotypic plasticity and the origins of diversity. Annu Rev Ecol Syst. 1989;20:249–78.

[7] Klingenberg CP. A developmental perspective on developmental instability: theory, models and mechanisms In: Polak M, editor. Developmental instability: causes and consequences: Oxford University Press; 2003 p. 14–34.

[8] Leary RF, Allendorf FW. Fluctuating asymmetry as an indicator of stress: implications for conservation biology. Trends Ecol Evol. 1989;4(7):214–7. 10.1016/0169-5347(89)90077-3 21227354

[9] Parsons P. Fluctuating asymmetry: an epigenetic measure of stress. Biol Rev. 1990;65(2):131–45. 2190634 10.1111/j.1469-185x.1990.tb01186.x

[10] Graham JH, Raz S, Hel-Or H, Nevo E. Fluctuating asymmetry: methods, theory, and applications. Symmetry. 2010;2(2):466–540.

[11] Klingenberg CP, Nijhout HF. Genetics of fluctuating asymmetry: a developmental model of developmental instability. Evolution. 1999;53(2):358–75. 28565420 10.1111/j.1558-5646.1999.tb03772.x

[12] Leamy LJ, Klingenberg CP. The genetics and evolution of fluctuating asymmetry. Annu Rev Ecol Evol Syst. 2005;36:1–21.

[13] Parsons P. Fluctuating asymmetry: a biological monitor of environmental and genomic stress. Heredity. 1992;68(4):361–4. 1563968 10.1038/hdy.1992.51

[14] Levin M. Left–right asymmetry in embryonic development: a comprehensive review. Mech Dev. 2005;122(1):3–25. 10.1016/j.mod.2004.08.006 15582774

[15] Kucharski R, Maleszka J, Foret S, Maleszka R. Nutritional control of reproductive status in honeybees via DNA methylation. Science. 2008;319(5871):1827–30. 10.1126/science.1153069 18339900

[16] Weaver IC, Cervoni N, Champagne FA, D'Alessio AC, Sharma S, Seckl JR, et al Epigenetic programming by maternal behavior. Nat Neurosci. 2004;7(8):847–54. 10.1038/nn1276 15220929

[17] Blewitt ME, Vickaryous NK, Paldi A, Koseki H, Whitelaw E. Dynamic reprogramming of DNA methylation at an epigenetically sensitive allele in mice. PLoS Genet. 2006;2(4):e49 10.1371/journal.pgen.0020049 16604157 PMC1428789

[18] Peyer B. Comparative odontology. Chicago: University of Chicago Press; 1968.

[19] Huysseune A, Sire JY. Evolution of patterns and processes in teeth and tooth‐related tissues in non‐mammalian vertebrates. Eur J Oral Sci. 1998;106(S1):437–81. 9541261 10.1111/j.1600-0722.1998.tb02211.x

[20] Engeman JM, Aspinwall N, Mabee PM. Development of the pharyngeal arch skeleton in *Catostomus commersonii* (Teleostei: Cypriniformes). J Morphol. 2009;270(3):291–305. 10.1002/jmor.10688 19034917

[21] Smith RE, Hocutt CH. Formulae variations of pharyngeal tooth counts in the cyprinid genus *Notropis*. Copeia. 1981;1981(1):222–4.

[22] Evans HE, Deubler EE. Pharyngeal tooth replacement in *Semotilus atromaculatus* and *Clinostomus elongatus*, two species of cyprinid fishes. Copeia. 1955;1955(1):31–41.

[23] Eastman JT, Underhill JC. Intraspecific variation in the pharyngeal tooth formulae of some cyprinid fishes. Copeia. 1973;1973(1):45–53.

[24] Essner JJ, Amack JD, Nyholm MK, Harris EB, Yost HJ. Kupffer's vesicle is a ciliated organ of asymmetry in the zebrafish embryo that initiates left-right development of the brain, heart and gut. Development. 2005;132(6):1247–60. 10.1242/dev.01663 15716348

[25] Qian M, Yao S, Jing L, He J, Xiao C, Zhang T, et al ENC1-like integrates the retinoic acid/FGF signaling pathways to modulate ciliogenesis of Kupffer’s vesicle during zebrafish embryonic development. Dev Biol. 2013;374(1):85–95. 10.1016/j.ydbio.2012.11.022 23201577

[26] Albertson RC, Yelick PC. Roles for *fgf8* signaling in left–right patterning of the visceral organs and craniofacial skeleton. Dev Biol. 2005;283(2):310–21. 10.1016/j.ydbio.2005.04.025 15932752

[27] Hubbs CL, Hubbs LC. Bilateral asymmetry and bilateral variation in fishes. Paper Mich Acad Sci Arts Lett. 1945;30:229–310.

[28] Stock DW. Zebrafish dentition in comparative context. J Exp Zool B Mol Dev Evol. 2007;308(5):523–49. 10.1002/jez.b.21187 17607704

[29] Pasco-Viel E, Charles C, Chevret P, Semon M, Tafforeau P, Viriot L, et al Evolutionary trends of the pharyngeal dentition in Cypriniformes (Actinopterygii: Ostariophysi). PLoS One. 2010;5(6):e11293 10.1371/journal.pone.0011293 20585584 PMC2892034

[30] Pasco-Viel E, Yang L, Veran M, Balter V, Mayden RL, Laudet V, et al Stability versus diversity of the dentition during evolutionary radiation in cyprinine fish. Proc R Soc B. 2014;281(1780):20132688 10.1098/rspb.2013.2688 24523268 PMC4027388

[31] Hung NM, Ryan TM, Stauffer JR, Madsen H. Does hardness of food affect the development of pharyngeal teeth of the black carp, *Mylopharyngodon piceus* (Pisces: Cyprinidae)? Biol Control. 2015;80:156–9.

[32] Dong G, Dong Q, Liu Y, Lou B, Feng J, Wang K, et al High-resolution micro-CT scanning as an innovative tool for evaluating dental hard tissue development. J Appl Clin Med Phys. 2014;15(4):335–44. 10.1120/jacmp.v15i4.4956 PMC5875498 25207426

[33] Leung C, Breton S, Angers B. Facing environmental predictability with different sources of epigenetic variation. Ecol Evol. 2016;6(15):5234–45. 10.1002/ece3.2283 27551379 PMC4984500

[34] Angers B, Schlosser IJ. The origin of *Phoxinus eos-neogaeus* unisexual hybrids. Mol Ecol. 2007;16(21):4562–71. 10.1111/j.1365-294X.2007.03511.x 17892466

[35] Binet MC, Angers B. Genetic identification of members of the *Phoxinus eos‐neogaeus* hybrid complex. J Fish Biol. 2005;67(4):1169–77.

[36] Elder JF, Schlosser IJ. Extreme clonal uniformity of *Phoxinus eos/neogaeus* gynogens (Pisces: Cyprinidae) among variable habitats in northern Minnesota beaver ponds. Proc Natl Acad Sci USA. 1995;92(11):5001–5. 7761438 10.1073/pnas.92.11.5001 PMC41835

[37] Goddard K, Megwinoff O, Wessner L, Giaimo F. Confirmation of gynogenesis in *Phoxinus eos-neogaeus* (Pisces: Cyprinidae). J Hered. 1998;89(2):151–7.

[38] Vergilino R, Leung C, Angers B. Inconsistent phylogeographic pattern between a sperm dependent fish and its host: *in situ* hybridization *vs* dispersal. BMC Evol Biol. 2016;16(1):1–12. 27600616 10.1186/s12862-016-0754-5 PMC5012089

[39] Palmer AR, Strobeck C. Fluctuating asymmetry as a measure of developmental stability: implications of non-normal distributions and power of statistical tests. Acta Zool Fenn. 1992;191:57–72.

[40] Demontis D, Pertoldi C, Passamonti M, Scali V. Increased fluctuating asymmetry in a naturally occurring hybrid zone between the stick insects *Bacillus rossius* rossius and *Bacillus rossius redtenbacheri*. J Insect Sci. 2010;10(1):147. 21070177 10.1673/031.010.14107 PMC3016888

[41] New JG. Hybridization between two cyprinids, *Chrosomus eos* and *Chrosomus neogaeus*. Copeia. 1962:147–52.

[42] Fedorov A, Beichel R, Kalpathy-Cramer J, Finet J, Fillion-Robin J-C, Pujol S, et al 3D Slicer as an image computing platform for the Quantitative Imaging Network. Magn Reson Imaging. 2012;30(9):1323–41. 10.1016/j.mri.2012.05.001 22770690 PMC3466397

[43] Jordan DS, Evermann B. The Fishes of North and Middle America. Bull U S Natl Mus. 1896;Pt. 1:(47):1–124.

[44] Zelditch ML, Swiderski DL, Sheets HD. Geometric morphometrics for biologists: a primer. San Diego: Elsevier Academic Press; 2012.

[45] Gunz P, Mitteroecker P. Semilandmarks: a method for quantifying curves and surfaces. Hystrix. 2013;24(1):103–9.

[46] Rohlf FJ, Slice D. Extensions of the Procrustes method for the optimal superimposition of landmarks. Syst Biol. 1990;39(1):40–59.

[47] Bookstein FL. Landmark methods for forms without landmarks: morphometrics of group differences in outline shape. Med Image Anal. 1997;1(3):225–43. 9873908 10.1016/s1361-8415(97)85012-8

[48] Mitteroecker P, Gunz P. Advances in geometric morphometrics. Evol Biol. 2009;36(2):235–47.

[49] Dryden I, Mardia K. Multivariate shape analysis. Sankhya Ser A. 1993;55:460–80.

[50] Rohlf FJ. Shape statistics: Procrustes superimpositions and tangent spaces. J Classification. 1999;16(2):197–223.

[51] Klingenberg CP, McIntyre GS. Geometric morphometrics of developmental instability: analyzing patterns of fluctuating asymmetry with Procrustes methods. Evolution. 1998;52:1363–75. 28565401 10.1111/j.1558-5646.1998.tb02018.x

[52] Adams DC, Collyer ML. A general framework for the analysis of phenotypic trajectories in evolutionary studies. Evolution. 2009;63(5):1143–54. 10.1111/j.1558-5646.2009.00649.x 19210539

[53] Collyer ML, Adams DC. Phenotypic trajectory analysis: comparison of shape change patterns in evolution and ecology. Hystrix. 2013;24(1):75–83.

[54] Collyer ML, Adams DC. Analysis of two‐state multivariate phenotypic change in ecological studies. Ecology. 2007;88(3):683–92. 17503596 10.1890/06-0727

[55] Borcard D, Legendre P, Drapeau P. Partialling out the spatial component of ecological variation. Ecology. 1992;73(3):1045–55.

[56] Peres-Neto PR, Legendre P, Dray S, Borcard D. Variation partitioning of species data matrices: estimation and comparison of fractions. Ecology. 2006;87(10):2614–25. 17089669 10.1890/0012-9658(2006)87[2614:vposdm]2.0.co;2

[57] Klingenberg CP, Barluenga M, Meyer A. Shape analysis of symmetric structures: quantifying variation among individuals and asymmetry. Evolution. 2002;56(10):1909–20. 12449478 10.1111/j.0014-3820.2002.tb00117.x

[58] Oksanen J, Blanchet FG, Kindt R, Legendre P, Minchin PR, O'Hara R, et al. vegan: Community Ecology Package. 2015:http://CRAN.R-project.org/package=vegan. (accessed August 2016)

[59] Adams DC, Otárola‐Castillo E. geomorph: an R package for the collection and analysis of geometric morphometric shape data. Methods Ecol Evol. 2013;4(4):393–9.

[60] Goddard KA, Dawley RM, Dowling TE. Origin and genetic relationships of diploid, triploid, and diploid-triploid mosaic biotypes in the *Phoxinus eos-neogaeus* unisexual complex In: Dawley R, Bogart J, editors. Evolution and ecology of unisexual vertebrates. New York State Museum, New York 1989 p. Bull. 466, pp. 268–80.

[61] Stasiak RH. Morphology and variation in the finescale dace, *Chrosomus neogaeus*. Copeia. 1977;1977(4):771–4.

[62] Eastman JT. The pharyngeal bones and teeth of Minnesota cyprinid and catostomid fishes: functional morphology, variation and taxonomic significance. [Ph.D. Thesis]: University of Minnesota.; 1970.

[63] Simpson EH. Measurement of diversity. Nature. 1949;163:688.

[64] Nijhout HF. Development and evolution of adaptive polyphenisms. Evol Dev. 2003;5(1):9–18. 12492404 10.1046/j.1525-142x.2003.03003.x

[65] Angers B, Castonguay E, Massicotte R. Environmentally induced phenotypes and DNA methylation: how to deal with unpredictable conditions until the next generation and after. Mol Ecol. 2010;19(7):1283–95. 10.1111/j.1365-294X.2010.04580.x 20298470

[66] DeWitt TJ, Sih A, Wilson DS. Costs and limits of phenotypic plasticity. Trends Ecol Evol. 1998;13(2):77–81. 21238209 10.1016/s0169-5347(97)01274-3

[67] Murren CJ, Auld JR, Callahan H, Ghalambor CK, Handelsman CA, Heskel MA, et al Constraints on the evolution of phenotypic plasticity: limits and costs of phenotype and plasticity. Heredity. 2015;115(4):293–301. 10.1038/hdy.2015.8 25690179 PMC4815460

[68] Roff DA. The evolution of threshold traits in animals. Q Rev Biol. 1996;71(1):3–35.

[69] Whitman D, Agrawal A. What is phenotypic plasticity and why is it important? In: Whitman D, Ananthakrishnan T, editors. Phenotypic Plasticity of Insects: Mechanisms and Consequences. Enfield, NH: Science Publishers; 2009 p. 1–63.

